# Development and Validation of a Clinical Prediction Model for Complicated Appendicitis in the Elderly

**DOI:** 10.3389/fsurg.2022.905075

**Published:** 2022-06-09

**Authors:** Hui Feng, Qingsheng Yu, Jingxing Wang, Yiyang Yuan, Shushan Yu, Feisheng Wei, Zhou Zheng, Hui Peng, Wanzong Zhang

**Affiliations:** ^1^ General surgery department, The First Affiliated Hospital of Anhui University of Chinese Medicine, Hefei, China; ^2^ Institute of Chinese Medicine Surgery, Anhui Academy of Chinese Medicine, Hefei, Anhui, China

**Keywords:** Complicated appendicitis, elderly, nomogram (Min5-Max 8), internal validation

## Abstract

**Background:**

For elderly patients with mild clinical symptoms of uncomplicated appendicitis(UA), non-surgical treatment has been shown to be feasible, whereas emergency surgical treatment is recommended in elderly patients with complicated appendicitis(CA), but it is still challenging to accurately distinguish CA and UA before treatment. This study aimed to develop a predictive model to assist clinicians to quickly determine the type of acute appendicitis.

**Methods:**

We retrospectively studied the clinical data of elderly patients with acute appendicitis who visited the First Affiliated Hospital of Anhui University of Traditional Chinese Medicine from January 2012 to January 2022. The patients were divided into UA group and CA group, and the general conditions, medical history, physical examination, laboratory examination and imaging examination were compared between the two groups, and SPSS 26.0 and R 4.0.2 software were used to establish CA clinic. Predict the model, and validate it internally.

**Results:**

The clinical data of 441 elderly patients with acute appendicitis were collected, 119 patients were excluded due to incomplete clinical data or other diseases. Finally, 332 patients were included in the study and divided into UA group (*n* = 229) and CA group (*n* = 103). By analyzing the clinical data of the two groups of patients, the duration of abdominal pain [OR = 1.094, 95% CI (1.056–1.134)], peritonitis [OR = 8.486, 95% CI (2.017–35.703))] and total bilirubin [OR = 1.987, 95% CI (1.627–2.426)] were independent predictors of CA (all *p *< 0.01). The model's Area Under Curve(AUC) = 0.985 (95% CI, 0.975–0.994). After internal verification by Bootstrap method, the model still has high discriminative ability (AUC = 0.983), and its predicted CA curve is still in good agreement with the actual clinical CA curve.

**Conclusion:**

We found that a clinical prediction model based on abdominal pain duration, peritonitis, and total bilirubin can help clinicians quickly and effectively identify UA or CA before treatment of acute appendicitis in the elderly, so as to make more scientific clinical decisions.

## Introduction

Acute appendicitis is one of the common causes of surgical acute abdomen in the elderly, and the incidence of appendicitis in the elderly has increased progressively over the past few decades, which may be related to an increase in average life expectancy and improvements in diagnostic techniques (1, 2). Acute appendicitis in the elderly has the characteristics of insidious onset, rapid progress and high mortality. In a large observational study of 164.579 patients with acute appendicitis, multivariate analysis showed that age greater than 65 years was a significant risk factor for death (3). In addition, the elderly often have respiratory, circulatory, endocrine and metabolic diseases, and their immune function declines. Once appendix perforation occurs, the risk of death is even greater (4). Studies have shown that the perforation rate of suppurative appendicitis is generally between 20% to 30%, but the incidence of perforation in elderly patients can be as high as 70%, the high risk of perforation may be due to luminal narrowing due to vascular stiffness and fibrosis in the appendix in elderly patients (5, 6). The mortality rate of elderly patients with perforation can reach 4% to 8%, which is significantly higher than 1% of patients without perforation (7, 8).

According to its clinical features and pathological changes, acute appendicitis can be divided into acute simple appendicitis, acute suppurative appendicitis, acute gangrenous appendicitis, perforated appendicitis and periappendiceal abscess. In the current classification, the first two types of appendicitis are called uncomplicated appendicitis (UA), while the last three are called complicated appendicitis (CA) (9). Surgical treatment is generally used for the treatment of elderly UA, and conservative treatment can also be tried in elderly UA patients with mild clinical symptoms who strongly wish to avoid surgery and accept the risk of recurrence (10). However, elderly CA has a high mortality rate and a poor prognosis, and surgery should be performed as soon as possible once CA is diagnosed in elderly patients (10). Therefore, early identification of CA, active surgical treatment and adequate preoperative preparation can reduce the incidence of intraoperative and postoperative complications and avoid the increase of the fatality rate, which is particularly important (11). Because the clinical symptoms of acute appendicitis in the elderly are not typical, it is easy to develop perforation and gangrene, and the increase in body temperature and white blood cells is not obvious, and emergency color Doppler ultrasound, CT and MRI cannot distinguish UA from CA. Therefore, based on the clinical manifestations of patients, laboratory Examination and imaging studies to diagnose CA remain a challenge (12–14).

At present, it is a good choice to use a clinical prediction model to distinguish UA from CA (15), but no relevant reports on the clinical prediction model of CA in elderly have been found by searching the literature. The rationale of clinical prediction model is to construct the nomogram of the logistic regression model, assign points to each influencing factor according to the contribution of each influencing factor in the model to the outcome variable, and then add up the scores to obtain the total score. Finally, through the functional transformation relationship between the total score and the probability of the outcome event, the predicted value of the individual outcome event was calculated. This retrospective study analyzed data on elderly patients with UA and CA, including demographics, clinical characteristics, laboratory tests, imaging studies, etc., to determine the association between risk factors and appendicitis. And using these data, we developed and validated a clinical prediction model for CA in the elderly. Through the clinical prediction model, clinicians can effectively distinguish UA and CA patients, so as to make more appropriate clinical decisions for elderly patients with appendicitis.

## Materials & Methods

### Study Population

The clinical data of elderly patients with acute appendicitis who visited the First Affiliated Hospital of Anhui University of Traditional Chinese Medicine from January 2012 to January 2022 were collected. Inclusion criteria: ① age ≥65 years old; ② pathological diagnosis of acute appendicitis after surgery (including laparoscopic surgery and open surgery); ③ no life-threatening cardiovascular and cerebrovascular diseases on admission; exclusion criteria: ① age <65 age; ② combined with periappendiceal abscess; ③ combined with major trauma and surgery history; ④ combined with blood system diseases, malignant tumors, mental diseases, etc; for periappendiceal abscesses, it is relatively easy to diagnose in clinical work, and after diagnosis, mainly with antibiotics with or without percutaneous tube drainage treatment, surgical treatment is rare, which is different from the principle of active surgical treatment of gangrenous or perforated appendicitis, so this study excluded periappendiceal abscesses. In this study CA specifically refers to acute gangrenous appendicitis and perforated appendicitis. The study was conducted in accordance with the Declaration of Helsinki (as revised in 2013). This study was reviewed and approved by the Ethics Committee of the First Affiliated Hospital of Anhui University of Traditional Chinese Medicine(2022MCZQ05). Informed consent for this retrospective analysis was waived.

### Pathological Assessment

According to postoperative pathological types, patients with acute appendicitis were divided into UA group and CA group. ① UA: Pathological types include simple and suppurative appendicitis, appearance of appendix swollen, serosa hyperemia, with or without purulent exudate on the surface; microscopically manifested as transmural inflammation, ulceration or hemorrhage, with or without extramural pus; ② CA: pathological types include gangrene and perforation, necrosis or partial necrosis of appendix wall, dark purple or black. Microscopically, there are signs of massive necrotic tissue or perforation in the outer wall of the appendix.

### Data Collection

This study is a retrospective study. The data of the general condition, medical history and physical examination, laboratory examination and imaging examination results of the patients were collected retrospectively using a structured case record form. The following clinical features were obtained: Age, gender, history of diabetes, abdominal pain duration(APD), shifiting pain in right lower quadrant, nausea or vomiting, temperature(TEMP), history of appendicitis, peritonitis, white blood cell count(WBC), neutrophil percentage(NEUT%), neutrophil count(NEUT), lymphocyte percentage(LY%), lymphocyte count(LY), neutrophil-to-lymphocyte ratio(NLR), percentage of monocytes, number of monocytes, red blood cell count(RBC), hematocrit, hemoglobin(HGB), platelets(PLT), prothrombin time(PT), activated partial thromboplastin time(APTT), alanine aminotransferase(ALT), Aspartate aminotransferase(AST), total bilirubin(TBil), creatinine(Cr), Ca^2+^, appendix diameter, appendix bezoar, periappendiceal fat infiltration, etc. It should be noted peritonitis means right lower quadrant tenderness or total abdominal tenderness, rebound tenderness, and abdominal muscle tension in patients with appendicitis. Because the ability of nerve conduction in elderly patients decreases with the increase of age, the pain sensation will also weaken, and the rebound sensitivity will decrease due to abdominal muscle atrophy. Therefore, the judgment of abdominal tenderness and rebound pain may be wrong in the elderly with appendicitis patients (15, 16). The abdominal muscle tension originates from the inflammatory stimulation of the parietal peritoneum, which is a relatively objective indicator. Therefore, in this study, whether abdominal muscle tension was used as the main indicator for judging peritonitis.

### Statistical Analysis

Statistical analysis was performed using SPSS 26.0 and R 4.0.2 software. The normally distributed measurement data is represented by means ± standard deviations, and the ratio between groups is represented by an independent sample *t* test; Non-normally distributed data were expressed as the median (lower quartile, upper quartile), and the Wilcoxon rank-sum test was used for comparison between groups. The enumeration data were expressed by the number of cases and percentages, and the comparison of unordered categorical data was performed by the *x*^2^ test. *p *< 0.05 considered the difference to be statistically significant.

The least absolute shrinkage and selection operator (LASSO) regression model was used to screen predictors. The screened predictors were included in multivariate logistic regression to analyze the independent predictors of CA. According to the analysis results, the nomogram of the logistic regression model for predicting CA was drawn by software R 4.0.2. The receiver operating characteristic curve of the model was developed to assess the discriminative power of the model. Calibration plot and Hosmer-Lemesshow test were used to evaluate the accuracy of the model; R 4.0.2 software was used to draw a decision curve analysis (DCA) plot to evaluate the clinical practicability of the model. The model was internally validated by applying the Bootstrap method to replicate 1,000 times.

## Results

### Baseline Characteristics of Patients Undergoing Appendectomy

**Figure 1:**
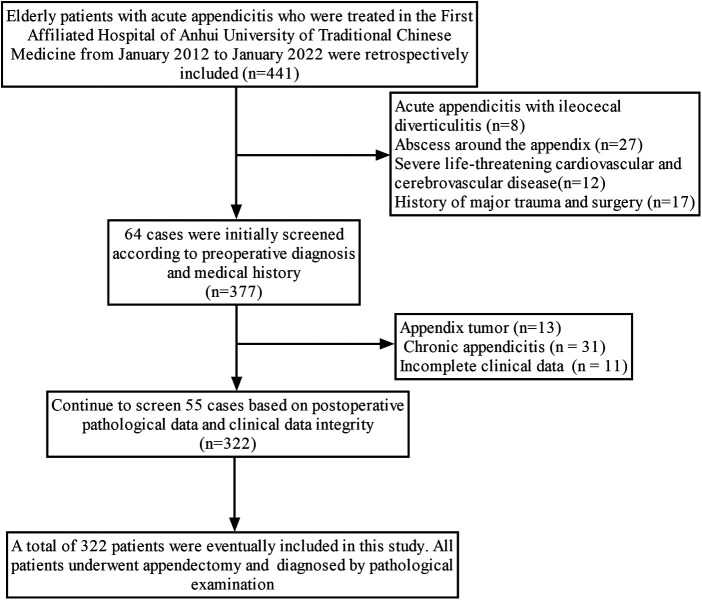
Flow chart of patient screening.

The clinical data of 441 elderly patients with acute appendicitis were collected, 119 patients were excluded due to incomplete clinical data or other diseases. Finally, 332 patients were included in the study and divided into UA group (*n* = 229) and CA group (*n* = 103) (suppl-Table 1). The patient screening process is shown in Figure 1. The baseline characteristics of UA (*n* = 229) and CA (*n* = 103) are summarized in Table 1. There were no significant differences in age, gender, history of diabetes, history of appendicitis, lymphocyte percentage (LY%), percentage of monocytes, number of monocytes, RBC, HGB, PLT, APTT, ALT, Cr, Ca^2+^, appendix diameter and appendix bezoar between the two groups (all *p *> 0.05). There were significant differences in the APD, shifiting pain in right lower quadrant, nausea or vomiting, TEMP, peritonitis, WBC, NEUT%, NEUT, LY, NLR, PT, AST, TBil and periappendiceal fat infiltration. (*Z *= −9.434, *x*^2^ = 7.846, *x*^2^ = 10.060, *Z *= −4.237, *x*^2^ = 50.790, *t = −*5.027, *Z *= −4.888, *Z *= −3.446, *Z *= −2.677, *Z *= −4.399, *t = −*2.729, *Z *= −3.119, *Z *= −13.097, *x*^2^ = 21.151, all *p *< 0.05).

**Table 1:** Baseline characteristics of patients undergoing appendectomy.

	Uncomplicated appendicitis (*n* = 219)	Complicated appendicitis (*n* = 103)	*x* ^2^ */t/Z*	*p* value
Age[y, median (Q1, Q3)]	71.00 (68.00,74.00)	72.00 (69.00,74.00)	*Z *= −1.764	0.078
Gender (%)			*x*^2^* = *1.110	0.292
Male, No. (%)	129 (58.9)	67 (65.0)	* *	
Female, No. (%)	90 (40.1)	36 (35.0)	* *	
History of diabetes, No. (%)	63 (28.8)	21 (20.1)	*x*^2^ = 2.551	0.110
APD [h, median (Q1, Q3)]	28.30 (15.90,42.40)	47.80 (38.45,62.35)	*Z *= −9.434	0.000
Shifiting pain in right lower quadrant, No. (%)	117 (53.4)	72 (69.9)	*x*^2^ = 7.846	0.005
Nausea or vomiting, No. (%)	101 (46.1)	67 (65.0)	*x*^2^ = 10.060	0.002
TEMP [°C, median (Q1, Q3)]	37.20 (36.90,37.60)	37.60 (37.00,38.40)	*Z *= −4.237	0.000
History of appendicitis, No. (%)	21 (9.6)	13 (12.6)	*x*^2^ = 0.682	0.409
Peritonitis, No. (%)	16 (7.3)	41 (39.8)	*x*^2^ = 50.790	0.000
WBC (×10^9^/L)	12.48 ± 3.35	14.55 ± 3.66	*t = −*5.027	0.000
NEUT% [%, median (Q1, Q3)]	81.30 (74.91,85.79)	85.67 (80.12,89.36)	*Z *= −4.888	0.000
NEUT [×10^9^/L, median (Q1, Q3)]	11.56 (9.11,13.20)	12.33 (10.20,15.45)	*Z *= −3.446	0.001
LY% [%, median (Q1, Q3)]	10.99 (6.58,15.81)	10.56 (7.08,13.19)	*Z *= −1.563	0.118
LY [×10^9^/L, median (Q1, Q3)]	1.50 (1.20,1.93)	1.38 (0.95,1.72)	*Z *= −2.677	0.007
NLR [median (Q1, Q3)]	7.18 (5.24,9.50)	9.45 (6.28,13.49)	*Z *= −4.399	0.000
Percentage of monocytes (%)	5.52 ± 1.90	5.22 ± 1.95	*t = *1.304	0.193
Number of monocytes [×10^9^/L, median (Q1, Q3)]	0.69 (0.44,1.03)	0.70 (0.42,0.88)	*Z *= −0.701	0.483
RBC (×10^12^/L)	5.00 ± 0.65	5.03 ± 0.69	*t = *−0.389	0.698
Hematocrit [%, median (Q1, Q3)]	41.37 (37.61,42.28)	40.59 (37.22,44.47)	*Z *= −0.234	0.815
HGB [g/L, median (Q1, Q3)]	133.00 (100.50,139.00)	123.00 (103.00,138.00)	*Z *= −1.007	0.314
PLT (×10^9^/L)	234.29 ± 56.65	234.81 ± 63.79	*t = −*0.073	0.942
PT (s)	12.43 ± 1.51	12.98 ± 1.79	*t = −*2.729	0.007
APTT (s)	31.27 ± 3.31	31.03 ± 3.11	*t = *0.236	0.537
ALT [U/L, median (Q1, Q3)]	30.00 (21.00,38.50)	33.00 (22.50,45.00)	*Z *= −1.934	0.053
AST [U/L, median (Q1, Q3)]	26.00 (17.00,35.00)	29.00 (21.50,40.00)	*Z *= −3.119	0.002
TBil [µmol/L, median (Q1, Q3)]	14.25 (11.89,16.90)	24.15 (21.42,27.13)	*Z *= −13.097	0.000
Cr [µmol/L, median (Q1, Q3)]	86.00 (79.00,101.50)	95.00 (75.00,104.00)	*Z *= −0.612	0.541
Ca^2+^ (mmol/L)	2.33 ± 0.12	2.31 ± 0.10	*t = *1.523	0.129
Appendix diameter (mm)	10.57 ± 3.54	11.25 ± 3.97	*t = *−1.490	0.138
Appendix bezoar, No. (%)	81 (40.0)	41 (39.8)	*x*^2^ = 0.236	0.627
Periappendiceal fat infiltration, No. (%)	39 (17.8)	43 (41.7)	*x*^2^ = 21.151	0.000

*Q1, lower quartile; Q3, upper quartile; APD, abdominal pain duration; TEMP, temperature; WBC,white blood cell count; NEUT%, neutrophil percentage; NEUT, neutrophil count; LY%, lymphocyte percentage; LY, lymphocyte count; NLR, neutrophil-to-lymphocyte ratio; RBC, red blood cell count; HGB, hematocrit, hemoglobin; PLT, platelets; PT, prothrombin time; APTT, activated partial thromboplastin time; ALT, alanine aminotransferase; AST, Aspartate aminotransferase; TBil, total bilirubin; Cr, creatinine.*

### Screening for Independent Predictors of CA

Using 10-fold cross-validation through LASSO regression, the Lambda value of 0.0309 corresponding to the minimum cross-validation error was taken as the optimal value of the model. From all the predictors, six potential risk factors, including APD, TEMP, peritonitis, NEUT%, NEUT, and TBil, were selected as predictors of CA. Multivariate logistic regression analysis showed that APD, peritonitis, and TBil were independent predictors of CA, as shown in Table 2.

**Table 2:** Multivariate logistic regression modeling.

Parameter	*β*	Wald *x^2^*	*p*-value	OR	95% CI
APD	0.090	0.018	<0.001	1.094	1.056-1.134
peritonitis	2.138	0.733	0.004	8.486	2.017-35.703
TBil	0.686	0.102	<0.001	1.987	1.627-2.426
Intercept	*−*15.520	2.272	<0.001	0.000	–

*APD, abdominal pain duration; TBil, total bilirubin; OR, Odds ratio; CI, confidence interval; “–” no value.*

### Development and Validation of Personalized Nomogram Prediction Model

**Figure 2:**
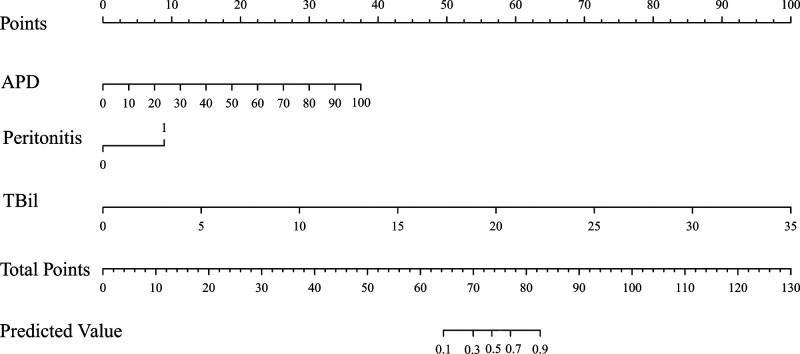
Nomogram for predictive complicated appendicitis model in the elderly. APD, abdominal pain duration; TBil, total bilirubin.

**Figure 3:**
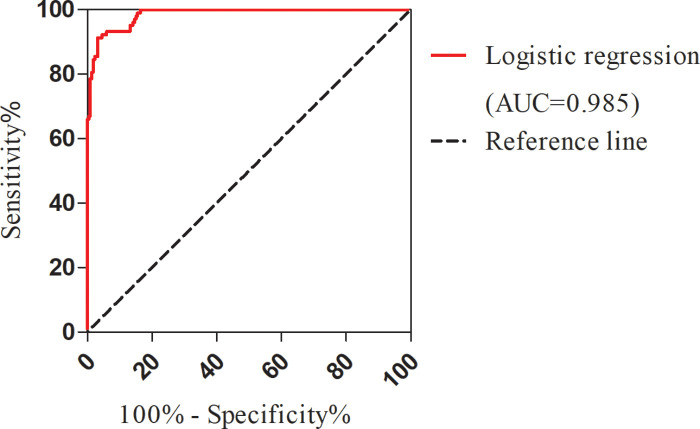
ROC curve for logistic regression models. ROC, receiver operating characteristic.

According to the multivariate logistic regression analysis results, the regression equation of the logistic regression model was obtained as logistic(*p*) = −15.520 + 0.09 × APD + 2.138 × peritonitis (1 for peritonitis, 0 for no peritonitis) + 0.686 × TBil. The model is presented in a nomogram, as shown in Figure 2. The area under the curve(AUC) of this model is 0.985 (95% CI, 0.975–0.994), as shown in Figure 3, AUC of APD is 0.826 (95% CI, 0.782–0.870), AUC of peritonitis is AUC = 0.662 (95% CI, 0.594–0.731), AUC of TBil was AUC = 0.952 (95% CI, 0.927–0.977).

### Evaluate the Accuracy of the Model

**Figure 4:**
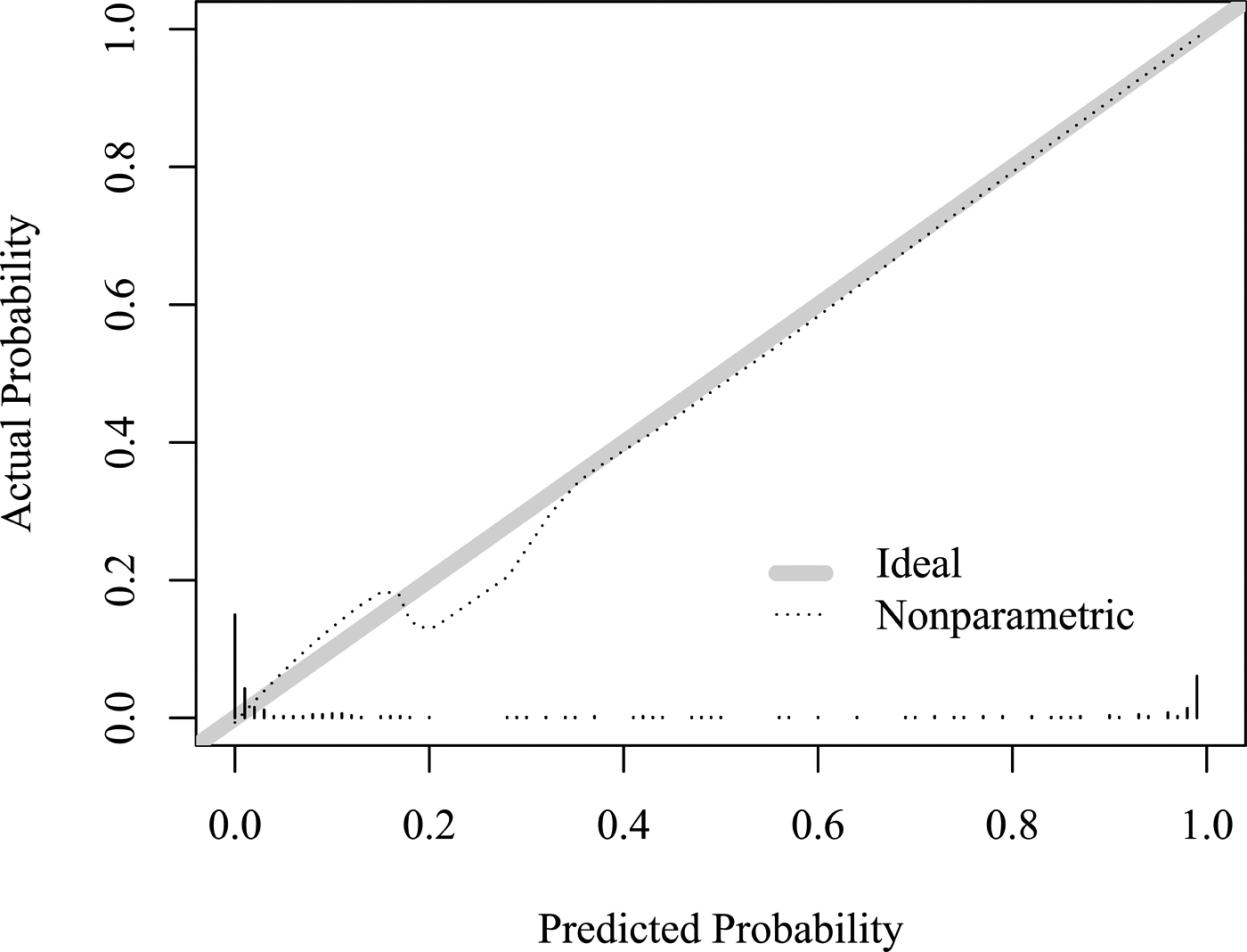
Model calibration diagram for predicting complicated appendicitis in the elderly.

In the Hosmer-lemeshow test, there was no statistically significant difference between the actual probability of CA and the predicted probability of CA (*x^2^* = 3.280, degrees of freedom = 8, *p *= 0.916). The calibration plot shows that the model prediction results are in good agreement with the actual clinical observations, as shown in Figure 4.

### Evaluate the Clinical Utility of the Model

**Figure 5:**
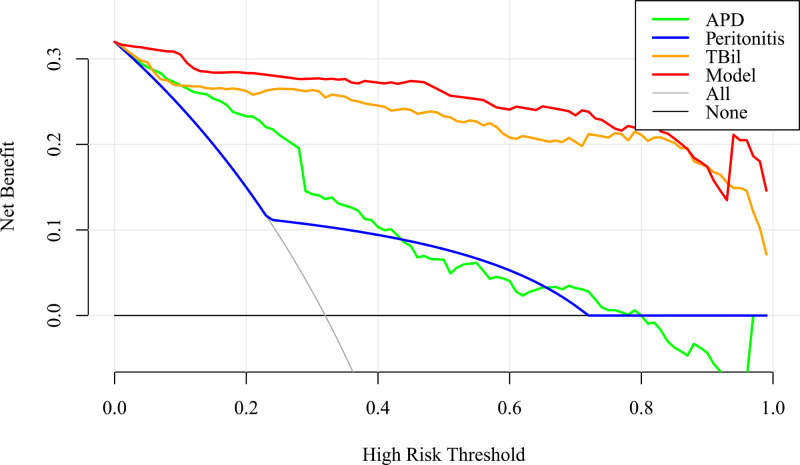
Decision curve analysis of the nomogram for complicated appendicitis in the elderly.

UA: uncomplicated appendicitis; CA: complicated appendicitis; None: the decision curve for not taking CA treatment plan assuming all UA patients are UA patients. All: the decision curve for assuming that all UA patients are take CA treatment plan; Model: the decision curve of the model; APD: the decision curve of APD; Peritonitis: the decision curve of peritonitis; TBil: the decision curve of TBil.

Compared with the decision curves of APD, Peritonitis, TBil and two extreme conditions, the decision curve of this model is basically higher than them (Figure 5), indicating that this model is relatively good. It can make patients benefit in the clinical practice and has certain clinical practical value.

### Internal Validation of the Model

**Figure 6:**
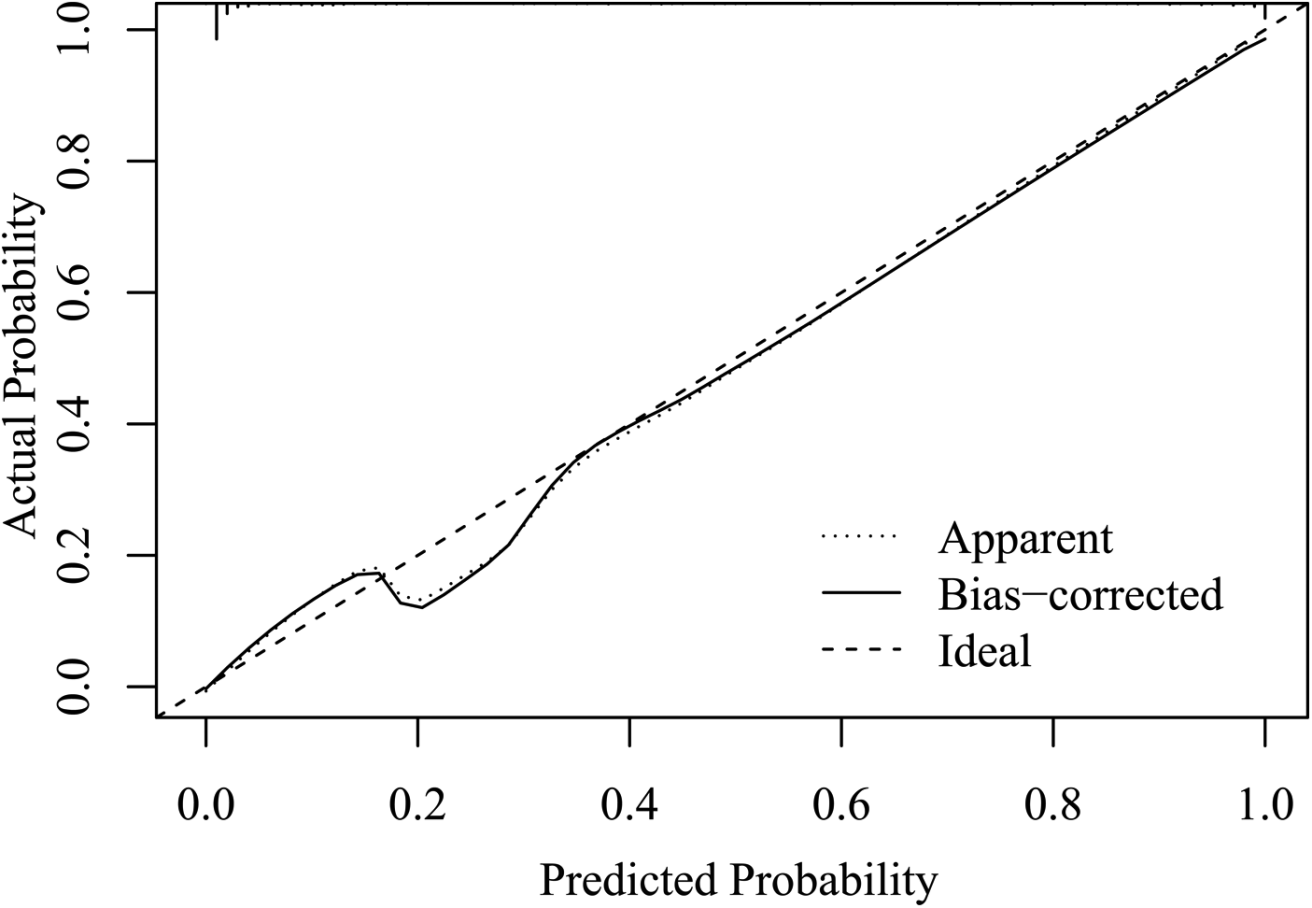
Calibration Plot of the Internally Validated Model for the Bootstrap Method.

After 1,000 internal verification and calibration by the Bootstrap method, the model still has a high discriminative ability (AUC = 0.983), and its predicted CA curve is still in good agreement with the actual clinical CA curve, as shown in Figure 6.

## Discussion

Once the diagnosis of CA in elderly patients is clear, surgery should be performed as soon as possible, otherwise life safety may be affected (10). However, how to predict CA early, quickly and accurately in elderly patients with appendicitis, so as to guide the clinical individualized treatment of elderly patients with appendicitis, has always been a difficult and hot topic in clinical research. With the development of precision medicine and personalized medicine, predictive models have gradually become a hot spot of clinical research in recent years (17). However, there is currently no clinical predictive model based on multiple predictors of elderly patients with CA. In this study, by retrospectively analyzing the case data of elderly patients with appendicitis, a predictive model that can be quickly and easily used clinically was established. The model will be verified internally, and the clinical practicability of the prediction model will be discussed in depth, in order to achieve early assessment of the condition of elderly patients with appendicitis and individualized treatment, so as to help clinicians make more scientific clinical decisions on treatment.

The disease of CA in the elderly progresses rapidly, and if the diagnosis and treatment are delayed, the prognosis of the patients will be seriously affected. Therefore, timely and accurate identification of CA is crucial for guiding clinical diagnosis and treatment. In this study, LASSO regression was used to screen out 6 potential risk factors for CA, including APD, TEMP, peritonitis, NEUT%, NEUT, and TBil among 30 candidate predictors. Multivariate logistic regression analysis showed that APD, peritonitis and TBil among the above 6 predictors were independent predictors of CA. Xu et al. showed that periappendiceal fat infiltration, C-reactive protein(CRP), and NLR were predictors of CA (17), which was different from our findings, possibly because their study population was adults, while our study population was elderly. However, the surgeons in our hospital failed to routinely detect CRP before surgery, so CRP was not included in our study.

APD is the time from the onset of abdominal pain to admission to the hospital. The study showed that APD before hospitalization in elderly patients with acute appendicitis was the most important risk factor for perforation, and the relative risk of perforation increased by 9% for each day of delay (RR: 1.09, *p* < 0.001) (18, 19). Imran JB et al reported that the median of APD in CA patients was longer than that in UA patients (2 days vs 1 day, *p *< 0.001), multivariate logistic regression analysis showed that APD was an independent predictor of CA (OR: 1.20; 95% CI, 1.07–1.37) (20). A recent study of CA patients in low- and middle-income countries also showed that the longer APD, the higher the incidence of CA, and surgeons should increase their awareness of the importance of APD in elderly patients (21, 22). This study found that APD in elderly patients with CA was significantly longer than that in patients with UA (CA vs. UA: 50.80 ± 13.37 vs. 30.31 ± 16.78, *p *< 0.001]). APD was an independent predictor of CA, and the regression coefficient was positive, indicating that the longer APD, the greater the possibility of developing CA, which was consistent with the above findings.

Peritonitis is a common and serious surgical disease caused by bacterial infection, chemical irritation or injury. Most of them are secondary peritonitis, originating from intra-abdominal organ infection, necrosis, perforation, trauma, etc. Peritonitis studied in this article refers to secondary peritonitis caused by gangrene or perforation of the appendix. Once peritonitis occurs in the elderly patients with appendicitis, it often indicates that the disease is more serious and the possibility of perforation is high. Neither UA nor CA is recommended for conservative treatment (10). Our long-term clinical experience also confirms this view. In conclusion, peritonitis is closely related to CA and can be used to predict the severity of appendicitis in the elderly.

Escherichia coli is the main pathogenic microorganism for appendicitis (23). Studies have found that Escherichia coli endotoxin can cause dose-dependent cholestasis, and Escherichia coli can also cause hemolysis of red blood cells, thereby increasing the bilirubin load (24, 25). Furthermore, in CA patients, severe inflammation can lead to intestinal edema and hypomotility, which can also lead to cholestasis (26). Sevinç MM et al found that TBil >1.0 mg/dL was significantly associated with appendix perforation (OR = 2.6) in 3392 patients with acute appendicitis (27). Eren T et al found that TBil >1.2 mg/dL was associated with gangrenous or perforated appendicitis (28). Motie MR et al believed that the incidence of hyperbilirubinemia in patients with gangrenous perforated appendicitis was higher than that of acute simple appendicitis (29). Although the association of hyperbilirubinemia with CA has long been known, however, this method is not widely used in daily clinical practice, Therefore, we suggest that early appendectomy should be considered in patients with hyperbilirubinemia and symptoms and signs of CA.

Three clinically accessible indicators were included in this study to construct the predictive model of CA, the AUC value of the model was 0.985, (95% CI, 0.975–0.994), indicating that the model has good discriminating ability for predicting CA. The model AUC value after Bootstrap internal validation was 0.983, and the calibration plot showed that the predicted curve was in good agreement with the actual observed curve. To demonstrate the utility of the model in the clinic, this study employed a novel approach, DCA, to inform clinical decisions based on threshold probabilities and to derive net benefit. The DCA plot shows that when the threshold probability is 0% to 100%, using this model to predict and identify CA and take corresponding treatment measures can benefit patients in clinical practice.

Limitations of this study: First, it was reported that CRP has a good predictive value in other clinical prediction models for acute appendicitis (30–32), but unfortunately, the surgeons in our hospital failed to routinely detect CRP before surgery, Therefore, CRP was not included in the analysis and will be included in future research to further improve the accuracy of the prediction model. Secondly, this study is a single-center retrospective study with a small sample size, and the model has not been verified externally. The accuracy of the model needs to be further verified by multi-center and large-sample studies.

In conclusion, the multivariate logistic regression analysis in this study concluded that APD, peritonitis, and TBil were independent predictors of CA in elderly patients. The clinical prediction model constructed based on these three indicators can predict CA in elderly patients with high accuracy. In the daily practice clinicians can incorporate the data (APD, peritonitis, and TBil) they obtain into our prediction model to calculate the predicted probability of a patient developing CA, which is helpful for clinicians to formulate more reasonable clinical plans, thereby saving medical expenses and improving patient prognosis.

## Data Availability

The original contributions presented in the study are included in the article/Supplementary Material, further inquiries can be directed to the corresponding author/s.
