# Supplementary material for: Development and Validation of a Clinical Prediction Model for Complicated Appendicitis in the Elderly
Source: Front Surg. 2022 Jun 9;9:905075. doi: 10.3389/fsurg.2022.905075 (PMC9218209; doi:10.3389/fsurg.2022.905075)
Supplement: Supplementary file 2 [file Table_2_v1.doc]

**Cover Letter**

Dear Editor:

The manuscript entitled “ Development and Validation of a clinical prediction model for complicated appendicitis in the elderly” was prepared following the Instructions for Authors of Qingsheng Yu.

In the present study, We found that a clinical prediction model based on abdominal pain duration, peritonitis, and total bilirubin can help clinicians quickly and effectively identify UA or CA before treatment of acute appendicitis in the elderly, so as to make more scientific clinical decisions.

The authors certify that they have participated sufficiently in the work to take public responsibility for the appropriateness of the experimental design and method, and the collection, analysis, and interpretation of the data. This manuscript has not been published in whole or in part nor is it being considered for publication elsewhere. The authors declare that there are no financial or other relationships that might lead to a conflict of interest of the present article. All authors have reviewed the final version of the manuscript and approved it for publication.

Now, the manuscript is submitting to Frontiers in Medicine and we look forward to hearing from you.

Yours sincerely,

Qingsheng Yu

The First Affiliated Hospital of Anhui University of Chinese Medicine, Hefei, 230031, PR, China

E-mail:ahszyyyqs@163.com
